# Everyone knows what science is…until you ask

**DOI:** 10.1098/rsos.260933

**Published:** 2026-11-11

**Authors:** Marina Dubova, Qiawen E. Liu

**Affiliations:** ^1^ Department of Psychology, University of California Berkeley, Berkeley, CA, USA; ^2^ Department of Computer Science, Princeton University, Princeton, USA

**Keywords:** metaphors of science, public understanding of science, scientific realism, conceptualizations of science

## Abstract

What is science? Scientists often assume that we share a general understanding of what science is, yet this assumption has rarely been empirically assessed. We examined scientists’ and members of the public’s agreement with a range of common conceptualizations of science, including science as a search for truth, a mirror of nature, a free marketplace of competing ideas and others. We found substantial heterogeneity in responses to these conceptualizations, with scientists disagreeing with each other more than the public does. We also found systematic differences in how the public and scientists understand science. Members of the public tended to endorse an idealized view of science, portraying it as a reliable machine for discovering objective truths through well-defined procedures. Scientists, in contrast, were more reserved in their endorsements, often emphasizing the limitations of the scientific method, contingency of scientific results and the roles of improvisation and creativity in scientific practice. This suggests that shared understanding of science cannot be taken for granted. Differences in how we understand science have implications for science practice, education and communication.

## 1. Introduction

*… what then is science? If no one asks me, I know; but if I want to explain it to a questioner I do not know.*—Max Charlesworth [1]

Science is full of disagreements [2]. Scholars have argued whether Earth orbits the Sun or vice versa, whether electrons are particles or waves, whether intelligence is innate or acquired and whether history is shaped by great individuals or broader social forces. Progress in science is often associated with articulating and resolving such disagreements [3–7].

While disagreeing about many things, researchers seem to operate under an unspoken assumption that we all agree on what science itself is [8,9]. Each of us specializes in a small aspect of the enterprise (e.g. some study cells, others black holes; some collect data, others build models), but we appear to act as though all these pursuits serve a shared activity called *science* that we all generally subscribe to. Examples of this are easy to find. Think about the last time you asked your colleague, *what is science to you*? Think about the class on scientific methods that you had to take or teach, was debating *what science is about* part of the class? When reading about best practices and crises in science, such as the replication crisis and its proposed solutions [10], how often is clarifying *what science is* part of the discussion? Even in public debates over science funding and its social value, how often do we ask whether scientists’ understanding of science aligns with that of the broader public?

From the authors’ personal experience of being trained and conducting research in cognitive science in the USA, these questions are rarely brought up. This experience may not be universal, but it is what motivated this study. We speculate that explicit conversations about science are rare because we all assume our understanding of science is a common ground, something so basic that does not need to be discussed (e.g. just like you probably never asked someone what they think a ‘table’ is, or whether they can smell ants; [11,12]). In debates among psychologists and cognitive scientists, for example, it is common to jump directly into specific scientific disagreements without clarifying the shared understanding of science itself. Another possible reason for this lack of discussion is that some may not think science is anything at all, or may assume, more pessimistically, that a conceptualization of science, if one exists, cannot be properly or usefully articulated [13–15].

This tendency to take ‘science’ as a given is not unique to practising scientists. Historians and sociologists of science have noted a similar lack of explicit discussion, even within disciplines devoted to studying science. As Steven Shapin observes, *‘Even historians and sociologists of science, on the occasions when they do consider the categories of science and the public, tend to focus upon the relations between two known entities, rather than analysing how the entities are themselves constituted’* [8,9,16]. Although some work examines how the boundaries of science are drawn and redrawn over time (e.g. [17,18]), much historical and sociological work treats science as a stable object of analysis, whose meaning is assumed rather than examined.

The relative absence of explicit conversations about what science is does not imply that possible differences in understanding are inconsequential. On the contrary, if scholars hold different conceptions of science, specific disagreements (e.g. on the correct methodology for a study, or a useful scientific question to address) may remain unsettled, because their source lies in divergent understandings of science itself. Moreover, systematic differences between how scientists and members of the public understand science could lead to persistent misalignment: scientists may fail to meet public expectations or needs, while the public may hold unrealistic views about what science can deliver and how it operates. Such misalignment may contribute to miscommunication and loss of trust in science.

We believe that differences in the understanding of science, if they exist, may act as hidden contributors to specific disagreements, both among scientists and between scientists and members of the public. Consider an example from Davis-Stober *et al.* [19], who reflect on disagreements about the rate and value of replication studies in science. Drawing on their own experience and conflicting literature, they suggest that a cognitive attitude (specifically, whether one thinks of the scientific literature as a ‘Book of Truths’ or a ‘Book of Conversations’) mediates both the diversity of scientists’ decisions about replication and the miscommunication between scientists and the public about non-replicable findings. Thus, latent disagreements about what science is and what its products should be may underlie more specific disagreements about which scientific questions to prioritize, which methodologies to choose, how to assess others’ work and how to communicate science results to the public.

Despite the high stakes of upstream misalignment about what science *is*, existing empirical work on public perceptions of science has largely focused on downstream attitudes towards science, such as trust in scientific results and advice [20], interest in different scientific topics [21] and knowledge of key scientific findings (i.e. science literacy [22,23]). In the body of work that *does* ask what science is—largely within science education—researchers typically evaluate students’, teachers’ and sometimes scientists’ views against a presumed ‘gold standard’ drawn from philosophical accounts of science, which is used to rank their level of science understanding [24–27]. Disagreement among experts themselves is rarely treated as a phenomenon of interest; instead, divergent views are typically interpreted as deviations from, or misunderstandings of, a single preferred conception of science rather than as reflecting a plurality of legitimate understandings. Notable exceptions come from experimental philosophy of science, where recent studies have surveyed scientists’ views on specific philosophical questions and documented systematic differences across fields [28,29].

### 1.1. Demarcation problem

The question of what makes science *science* has been discussed in philosophy as the demarcation problem: what distinguishes sciences, like biology, chemistry and physics, from non-sciences, like astrology and alchemy [1,30]? Decades of philosophical work have shown this question to be very difficult to answer [31]. Different criteria have been proposed, such as the idea that scientific statements are verifiable (i.e. can be corroborated by evidence [32,33]), falsifiable (i.e. can be disproven by evidence [5,34]) or that science is characterized by local problem-solving within theoretical paradigms [3,35]. Others have suggested that science is defined by a set of epistemic norms, such as scepticism towards all claims and an impersonal attitude to truth [36]. All of these criteria, however, tend to run into exceptions: they do not always match what scientists actually do [6], and famous scientific advances have been achieved without close attention to verifiability, falsifiability, the expected epistemic norms or other proposed criteria. This led Feyerabend to suggest that ‘anything goes’ in science [37].

The difficulty of defining science, at least in a way that scientists and philosophers would agree on, has led to sociological and empirical analyses of science, which aim to capture what scientists actually do (‘science is as science does’) and what authority science has in society [38,39]. Many philosophers and historians of science have begun to use surveys and other descriptive methods to learn what science is in practice [40–43], rather than asking what criteria should, in principle, justify science’s special role in society. These analyses have highlighted the diversity of practices, conceptualizations, cognitive attitudes, values and goals that characterize scientists within and between fields [6,28,42,44]. Other work has suggested that scientists draw and redraw the boundaries of science in an effort to create a public image of science that separates it from pseudoscience [17,18]. Science, then, may not be a single entity, and different scientists may understand it in different ways.

### 1.2. Is science definable?

Coming up with a clear definition of science may be exceptionally difficult, for reasons that are more general than the complexity of science itself. Psychologists studying human concepts have found that, in general, we do not learn and use concepts that fit black-and-white definitional schemes. Instead, our concepts seem to be characterized by fuzzy boundaries (there are many edge cases and exceptions [14,45]), graded structure (there are better and worse members of a concept [46]) and diversity (people who share a culture and native language understand the same concepts in very different ways [47]). Even our simplest concepts turn out not to be strictly definable: the concept ‘bird’, however you define it, has to accommodate many exceptions, like penguins and emus. Such concepts also have graded structure: a robin is a better instance of a bird than a penguin is [46]. The dominant view in the psychology of concepts is thus to think of concepts as fuzzy rather than easily definable [45], and it would be unreasonable to expect that a concept as complex as science would somehow circumvent this basic feature of human conceptualization.

Here, we use metaphors to study how scientists and members of the public conceptualize science. This method allows us to tap into the rich and fuzzy conceptual structure that a definitional approach may not reach.

Because our approach, analyses and interpretations inevitably reflect our own understanding of science, we briefly articulate it here. First, we think that precise articulation is exactly what is often impossible for science, which is why we chose a metaphor-elicitation approach. As cognitive scientists, we do not take science to be definable by a single criterion. We view it as a diverse, socially organized human activity in which researchers actively interact with phenomena and create and revise representations of the world. These representations are constrained by evidence but are also shaped by non-epistemic factors such as scientists’ cognitive biases, values and historical circumstances. Science is messy, yet it somehow seems to make progress, at least sometimes. Our perspective is therefore closer to active, haptic and perspectival realist accounts (e.g. [48–52]) than to a view that science provides an objective reflection of reality.

### 1.3. Metaphors as probes of conceptual structure

Metaphors provide an indirect but powerful way to probe how people think about a concept. When people interpret a metaphor, they project relational structure from a familiar source domain onto a target domain. This highlights some aspects of the target over others and supports particular inferences, or entailments, about it [53,54]. For example, describing crime as a beast rather than a virus led people to propose more enforcement-oriented rather than reform-oriented solutions to crime, consistent with the different inferences suggested by the two source domains [55]. Metaphors can therefore reveal which properties and relations people see as relevant to a target concept (see [56] for a review). Science, similarly, is an abstract concept that can be understood or represented through different metaphors. Construing science as reading a book of nature can entail the idea that information is already present in the world and awaits interpretation or discovery [57]; construing science as construction work instead highlights scientists’ active role in building and revising knowledge [58].

The interpretations of a metaphor are constrained but not uniquely determined, the same metaphor can support several coherent relational mappings [59,60]. Some metaphors may invite a relatively narrow set of entailments (e.g. a mirror highlights reflection and correspondence with an external reality), some metaphors are more open-ended (e.g. a game may evoke play, engagement, rules, goals, competition, strategy or chance). However, such flexibility in how metaphors are interpreted does not imply that metaphor endorsement is uninformative. If distinct metaphors that support overlapping entailments tap into common aspects of how science is conceptualized, endorsement of those metaphors should covary across respondents and give rise to coherent higher-order structure. On the other hand, if responses mainly reflect idiosyncratic interpretations of individual metaphors, such convergence should be minimal. We therefore examine patterns of covariation across metaphors, together with participants’ explanations where available, to assess the extent to which different framings capture shared or divergent ways of thinking about science.

Here, we probe how members of the public and scientists—across disciplines ranging from physics to history and across career stages from master’s students to Nobel laureates—react to common conceptualizations of science, identifying points of disagreement among scientists and the principal dimensions along which scientists’ and the public’s views diverge.

## 2. Assessing conceptualizations of science

We collected responses from scientists (*N* = 92) and members of the public (*N* = 99^1^) asking them to assess, on a 7-point Likert scale,^2^ their agreement with a set of conceptualizations of science. These conceptualizations were drawn from the history and philosophy of science and from public discourse about science (table 1). They included describing science as ‘mirroring nature’ [68] or ‘reading a book of nature’ [57] (visual), thinking of science as the ‘cumulative construction of knowledge, brick by brick’ [58] or as ‘a craft carving nature at its joints’ [62] (crafts), conceptualizing science as a ‘free marketplace of competing ideas’ [81] (economic) or as an ongoing ‘battle against ignorance’ [86] (battle), among others. Some of these conceptualizations have a long history (e.g. ‘science as carving nature at its joints’, which traces back to Plato), whereas others are relatively modern (e.g. science as a ‘free marketplace of ideas’, associated with economic and political developments from the nineteenth century onward). We selected the conceptualizations by reviewing the literature on analogies that have been used to describe science (see sources in table 1). Some are based on scientists’ own descriptions of their work, while others come from historians and philosophers of science analysing scientific method; some describe what it is like to do science, whereas others describe what science should do. The list is not intended to be comprehensive.

**Table 1.** Conceptualizations of science that participants were probed with (left), example sources of the probed conceptualizations (middle) and example reasons our scientist respondents disagreed with them (right).

probed conceptualization	example source	example disagreement quote from a scientist
Science is like construction work (i.e. building knowledge brick by brick, filling in the knowledge gaps).	*Science is built up with facts, as a house is with stones. But a collection of facts is no more a science than a heap of stones is a house.*—H. Poincaré [61]	*Certainly, until someone arrives with a bulldozer to gentrify the neighborhood.*—PhD student, history and philosophy
Science is a craft that reveals natural divisions (e.g. carving nature at its joints).	*Like an animal, the world comes to us predivided. Ideally, our best theories will be those which ‘carve nature at its joints.’*—Plato, Phaedrus 265e (fourth century BCE) [62]	*There are chances many of these natural divisions are human constructs. I’m not sure nature really cares about natural kinds.*—PhD student, physics
Doing science is like reading a book of nature.	*Reading a large book on the nature of things* —Augustine of Hippo, Contra Faustum, XXXII, 20 (fourth century AD) [57,63,64]	*That’s poetic but again reading a book seems too passive. it also suggests that nature is a constant and unchanging entity that can be captured in a book, rather than a dynamic system undergoing constant change*—Professor, biological sciences
Science is like a flashlight, casting light on obscurities.	*Science as a Candle in the Dark* [65], Enlightenment, Light as truth [66]	*I would opt for the photo negative of the analogy. I get the impression that, from a psychological perspective, the pre-scientific world was a world of light, where people and things had their place. Science came along and introduced obscurity, complexity, and confusion in all sorts of unexpected places. I guess it cleared some things up too :)*—PhD student, aerospace engineering
Science is like a mirror aiming to reflect reality.	The ‘Great Mirror’ (with its subvolume ‘Mirror of Nature’), written in the thirteenth century, is the biggest European encyclopedia of knowledge of that time [67]. *How Science Reflects Reality* [68]	*I think of it as a probe more than a mirror. Mirror is too passive and kind of uninteresting. Why use a mirror if you can just look.*—Professor, biological sciences
Science is like detective work, solving mysteries.	*I used the metaphor of a detective to explain my work. As paleontologists, we study the past like detectives and look for clues in the ground.*—F. Bulian, quoted in [69]	*For some (many?) scientific questions we don’t know if there is a solution, and often there may be alternative ‘solutions’ to a problem.*—Professor emeritus, geological sciences
Science is like a game.	*You can’t play 20 questions with nature and win* [70]. *The chessboard is the world; the pieces are the phenomena of the universe; the rules of the game are what we call the laws of Nature*—T. Huxley [71]	*It can be fun, but I wouldn’t say it’s as interactive.*—Postdoc, computer science
Science is like creative improvisation (e.g. improv).	Improvisational science: *To be creative, you have to open up and improvise, also in science.*—I. Yanai & M. Lercher [72].	*I see some connections between improv and some parts of the scientific process (e.g. brainstorming, thought experiments), but most of what I see as science is radically different from improv (more cognitive focus, rigour, possibility of being ‘wrong, and a lot more)*—PhD student, management
Doing science is resolving specific problems within paradigms (e.g. puzzle-solving).	*So long as the tools a paradigm supplies continue to prove capable of solving the problems it defines, science moves fastest…*—T. Kuhn [3]. *The aim of science is merely to secure theories with a high problem-solving effectiveness.*—L. Laudan [73]	*This may be what the day-to-day work of most scientists is. I don’t think it’s necessarily the ideal, nor is it the complete story. We all want to be revolutionaries.*—Professor, environmental social sciences
Doing science is like touching reality (i.e. interacting with phenomena through measurement instruments that can shape the phenomena themselves).	Science should strive to maximize our contact with reality [48,49]. Haptic realism: *the acquisition of scientific knowledge is an active process in which the scientist’s schematization and the work that goes into shaping the material target of research leave an indelible imprint on scientific knowledge.*—M. Chirimuuta [50,74]	*It makes it sound like science is mostly ‘doing.’ I believe science is mostly ‘thinking, not ‘doing.’*—Professor, management
Science is a search for beauty and elegance.	*One thing that makes the adventure of working in our field particularly rewarding, especially in attempting to improve the theory, is that…a chief criterion for the selection of a correct hypothesis…seems to be the criterion of beauty, simplicity, or elegance.*—M. Gell-Mann [75]	*Beauty and elegance are cognitive limitation to doing science. They are insurmountable obstacles not goal.*—PhD student, psychology
Science is a search for truth.	*Science is the search for the truth*—L. Pauling [76]	*I don’t know what truth is and I don’t think anyone else does either.*—PhD student, aerospace engineering
Science is an iterative dialogue with nature.	*What we observe is not nature itself, but nature exposed to our method of questioning*—W. Heisenberg [77]	*In many research, it’s much more about the science doing a monologue, and us trying to understand what it says. Most often, we don’t answer back.*—Postdoc, computer science
Doing science is creating simplified representations of the world (e.g. a map of complex reality).	*On exactitude in science*—J. L. Borges [78]; *All models are wrong, some are useful.*—G. Box [79]	*The map can be as complex as the territory…*—Professor, humanities
Science is a curiosity-driven exploration.	*…we deal with curiosity-driven science, that is, research that is driven by the curiosity of scientists who select for themselves which research questions and methods of investigation they wish to pursue*—Gigerenzer *et al.* [80]	*This is an ideal, sure. I don’t think it’s what many practicing scientists are doing though.*—Professor, anthropology
Science is a free marketplace of competing ideas.	*The republic of science: its political and economic theory* [81,82]	*It’s not free (there are massive barriers to entry). And who knows what ideas are the most successful?*—Postdoc, biology
When doing science, we construct reality instead of discovering it.	Constructivism [83]; performativity, reflexivity, looping effects [84,85]	*We construct theories; reality is already there.*—Professor, philosophy
Science is a battle against ignorance.	*There are in fact two things, science and opinion; the former begets knowledge, the latter ignorance.*—Hippocrates	*I strongly disagree because currently science is a direction of exploring the world which excludes many other ways of exploring it. Thus as one advances in science right now i believe one is more and more ignorant to other ways of understanding and learning.*—PhD student, mathematics

Participants were also invited (scientists) and required (public) to explain their judgments.^3^

Public respondents were recruited via the Prolific platform using the platform’s standard (non-representative) recruitment, restricted to US residents. Participants were paid at the rate of $12 per hour. The copy-and-paste feature was disabled to reduce the likelihood of AI-generated responses (one participant nevertheless produced such responses and was excluded, based on responses that exceeded twice the length of all other participants, which did not pass the AI checker). The average age of public participants was 45 years; 52% identified as female, 47% as male and 1% as non-binary. All public participants resided in the USA, nearly all were US-born (98%), and all spoke English as a first language. According to Prolific’s demographic records, 77% identified as White, 12% as Black, 4% as mixed, 3% as Asian and 4% as another ethnicity. Among participants with current employment records (n=80), 50% worked full time, 16% part time, 14% were unemployed and seeking work and 16% were not in paid work.

To obtain a sample spanning multiple scientific fields and career stages, participants in the scientists’ group were recruited primarily from individuals visiting the interdisciplinary Santa Fe Institute (91% of participants) and from the SciFoo academic camp mailing list (9%). The sample included scientists from a wide range of disciplines: 23% biological sciences, 17% psychological and cognitive sciences, 16% physics, 13% social sciences, 11% humanities and arts and philosophy, 9% engineering and computer science, 5% mathematics, 4% earth and environmental sciences and 1% other. The sample was also diverse in career stage and gender (career stage: 2% master’s students, 26% PhD students, 20% postdoctoral researchers, 10% research scientists, 4% assistant professors, 10% associate professors, 20% full professors, 8% emeritus professors and 1% other; gender: 30% female, 68% male and 2% non-binary; average age: 41). This study was approved by the Institutional Review Board of Indiana University Bloomington (protocol no. 25815, ‘Metaphors of Science’). All participants provided informed consent before taking part.

## 3. (Dis)agreement in science understanding

Within and between groups, individuals disagreed on many conceptualizations of science (figure 1). Here, we examine the degree of disagreement among scientists, the degree of disagreement among members of the public, the degree of disagreement between these groups and the dimensions that organize these disagreements.

**Figure 1.**
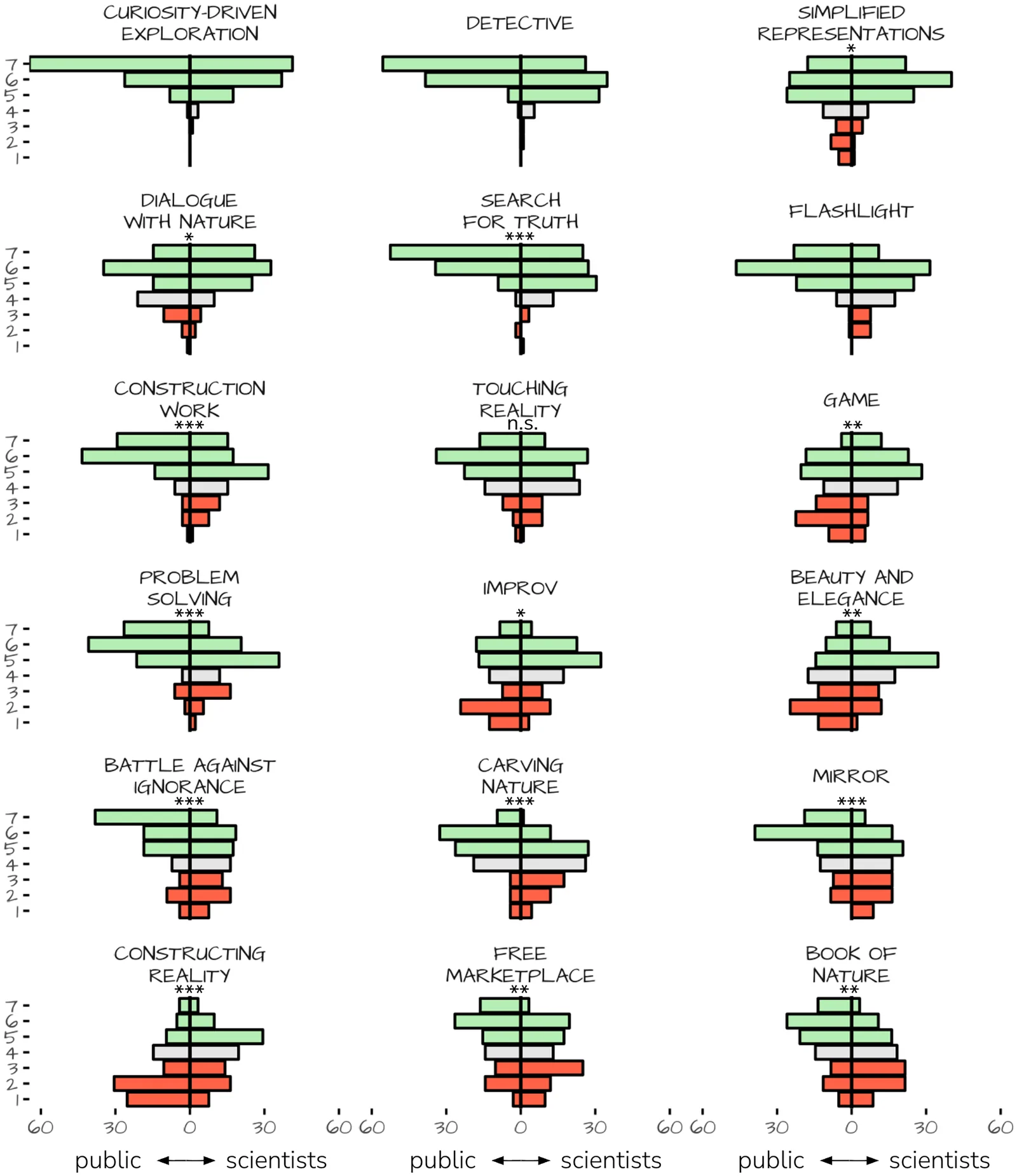
Agreement distributions for different probed conceptualizations of science, shown separately for members of the public (left) and scientists (right). Responses ranged from 1 (strongly disagree) to 7 (strongly agree). Conceptualizations are ordered by scientists’ mean agreement score (from highest to lowest, left to right, top to bottom). Asterisks indicate statistical significance of differences between the public and scientist response distributions, assessed using chi-square tests and corrected for multiple comparisons (supplementary material, table S1). Note that for three conceptualizations (curiosity-driven exploration, detective work and flashlight), nearly all participants in both groups agreed, leaving one or more response categories empty in both groups; chi-square tests are undefined for these cases.

First, we assessed responses *within* groups of participants, quantifying how much scientists agree with scientists and members of the public agree with members of the public. For this, we computed the mean pairwise correlation between participants’ item-response profiles, separately for members of the public and scientists. We conducted parallel analyses using Pearson and Spearman correlation coefficients (i.e. treating Likert responses as approximately continuous versus ordinal). Members of the public showed a relatively high degree of shared structure in their response profiles (Pearson: 0.37; Spearman: 0.37; permutation test p<0.001 for both), whereas scientists exhibited a lower, but still clearly non-random, level of agreement (Pearson: 0.23; Spearman: 0.24; permutation test p<0.001 for both). A permutation test further indicated that the public’s agreement was significantly higher than that of scientists (p=0.002 for both measures). Thus, scientists disagreed more among themselves about what science means than members of the public did (figure 2).

**Figure 2.**
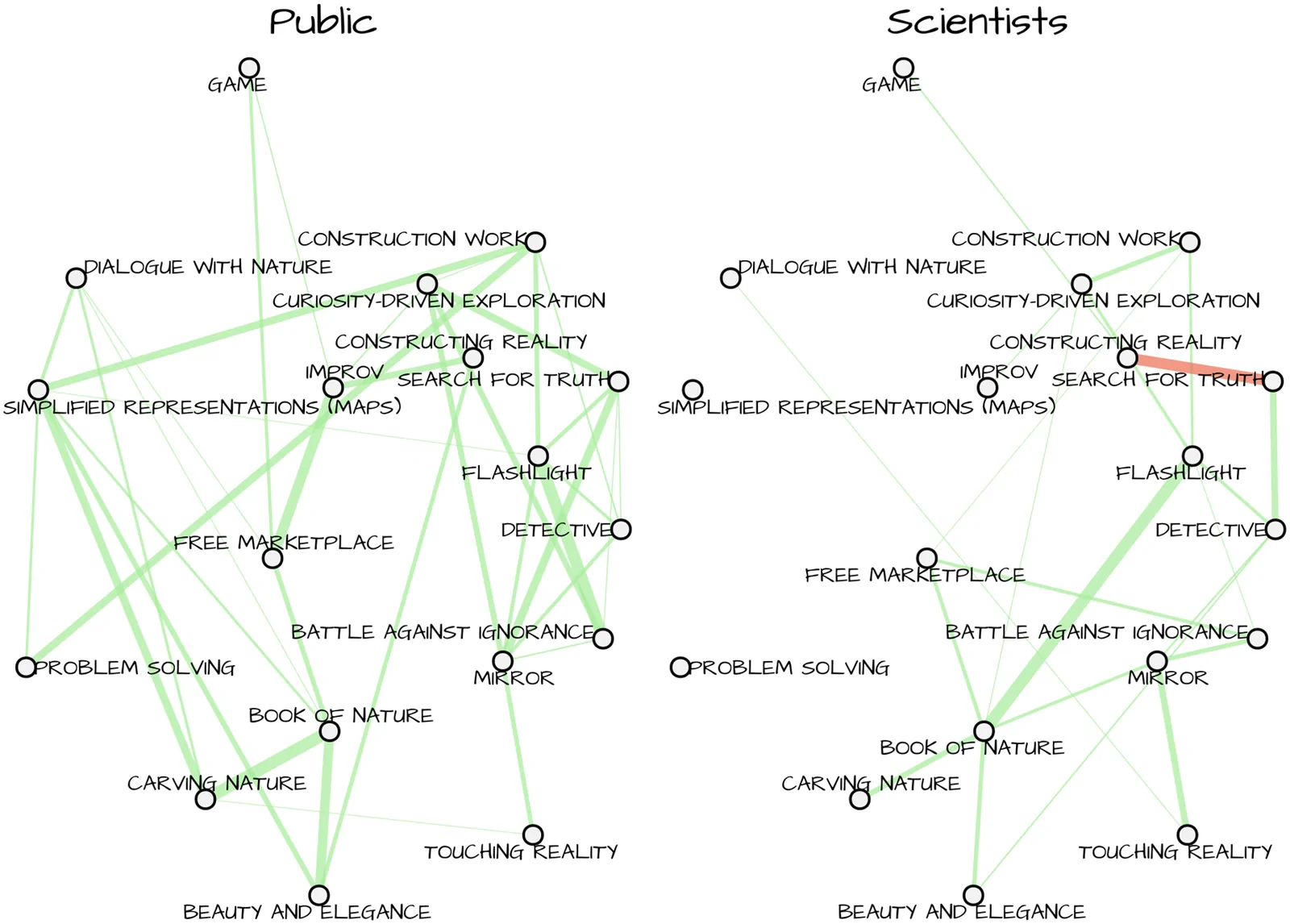
Network of correlations among responses to different questions for the public (left) and scientists (right). Networks were constructed using Spearman correlations with a threshold of greater than 0.25 or less than −0.25. Green edges indicate positive correlations (answering one question positively is associated with answering another question positively), and red edges indicate negative correlations. The public network is more densely connected, whereas the scientists’ network is sparser, suggesting greater heterogeneity in scientists’ response patterns. Only the colour and strength of the edges should be interpreted. Node positions carry no meaning along the x- or y-axes: they were chosen to make the edges easy to see and are held fixed across the two groups to simplify comparison.

Then, we looked more closely into disagreements *between* scientists and the public. For each conceptualization, we tested whether the distribution of ratings (from 1 = strongly disagree to 7 = strongly agree) differed between scientists and the public using chi-square tests of independence, controlling false discovery rate via the Benjamini–Hochberg procedure [87] (figure 1; supplementary material, table S1).

For three conceptualizations (curiosity-driven exploration, detective work and flashlight), nearly all participants in both groups agreed, leaving several response categories empty in both groups. Chi-square tests are undefined in this case, because empty categories have expected counts of zero. We interpret these cases as reflecting shared, widely endorsed conceptualizations of science.

Remaining comparisons revealed systematic differences between scientists and the public for most conceptualizations of science, with 14 of 15 testable conceptualizations showing significant group differences, with moderate effect sizes (Cramér’s V = 0.29–0.43). The largest differences concerned metaphors framing science as a search for truth, a mirror of nature, construction work and problem-solving within paradigms.

Consistently, principal component analysis (PCA) revealed two dominant and conceptually interpretable axes of variation (PC1 explaining 21% of the variance and PC2 explaining 12%; see table 2 for full loadings). Nearly all conceptualizations loaded positively on the first principal component, with the highest loadings for metaphors framing science as revealing or discovering an existing reality—science as a flashlight (0.36), a mirror (0.33), detective work (0.31), reading the book of nature (0.30) and a search for truth (0.30). The view that science constructs reality loaded negatively (−0.13) on this dimension. We therefore interpret PC1 as an endorsement of a realist conceptualization of science.^4^ The second principal component distinguished framings that emphasize creative, playful and aesthetically guided inquiry from conceptualizations that prioritize truth-seeking and accurate mirroring of the world. PC2 loaded highly on conceptualizations emphasizing improvisation (0.45), constructing reality (0.43), a game (0.40), beauty and elegance (0.38) and simplified representations (0.34), as opposed to science as a search for truth (−0.20). Together, these two components accounted for approximately 33% of the total variance, indicating that while they capture major dimensions of disagreement, substantial variation remains distributed across additional, orthogonal dimensions.

**Table 2.** Loadings of each conceptualization on the first two principal components (PC1: 21% of variance and PC2: 12%). Loadings with absolute value greater or equal to 0.30 are shown in bold.

conceptualization	PC1	PC2
flashlight	**0.36**	−0.08
mirror	**0.33**	−0.08
detective work	**0.31**	−0.06
book of nature	**0.30**	0.15
battle against ignorance	**0.30**	−0.04
search for truth	0.30	−0.20
curiosity-driven exploration	0.28	0.04
construction work	0.27	0.07
free marketplace	0.26	0.14
problem-solving within paradigms	0.24	−0.05
touching reality	0.21	0.05
carving nature at its joints	0.21	0.07
dialogue with nature	0.08	0.29
beauty and elegance	0.07	**0.38**
simplified representations (maps)	0.06	**0.34**
improvisation	0.04	**0.45**
game	−0.04	**0.40**
constructing reality	−0.13	**0.43**

**Table 3.** Alignment between scientists’ response profiles and the public average response profile by field (Pearson correlations). Values represent mean correlations, with 95% bootstrap confidence intervals obtained by resampling participants within each field. One person was excluded because of the unique field (complex systems).

field	n	mean	95% CI
biological sciences	21	0.417	(0.318, 0.507)
physics	15	0.384	(0.280, 0.491)
earth and environmental sciences	4	0.350	(0.108, 0.559)
engineering and computer science	8	0.277	(0.065, 0.476)
humanities and arts and philosophy	10	0.249	(0.111, 0.388)
psychological and cognitive sciences	16	0.207	(0.103, 0.306)
math	5	0.207	(0.056, 0.395)
social sciences	12	0.179	(0.065, 0.306)

Interestingly, the two groups (scientists versus members of the public) occupied systematically different regions of the two-dimensional space (figure 3). A logistic regression classifier based on participants’ scores on the two principal components predicted group membership with 82% leave-one-out cross-validated accuracy. Members of the public tended to view science as a way to obtain true facts (higher on ‘realism’) by following well-defined procedures (lower on ‘creativity’). Scientists, by contrast, were less likely to endorse strong realist conceptions of science and more likely to resonate with its playful, improvisational and creative aspects. This difference may partly reflect the composition of our scientist sample. It consisted mainly of visitors to the Santa Fe Institute, who are often selected for their engagement in interdisciplinary, creative research and may therefore differ from the average scientist.

**Figure 3.**
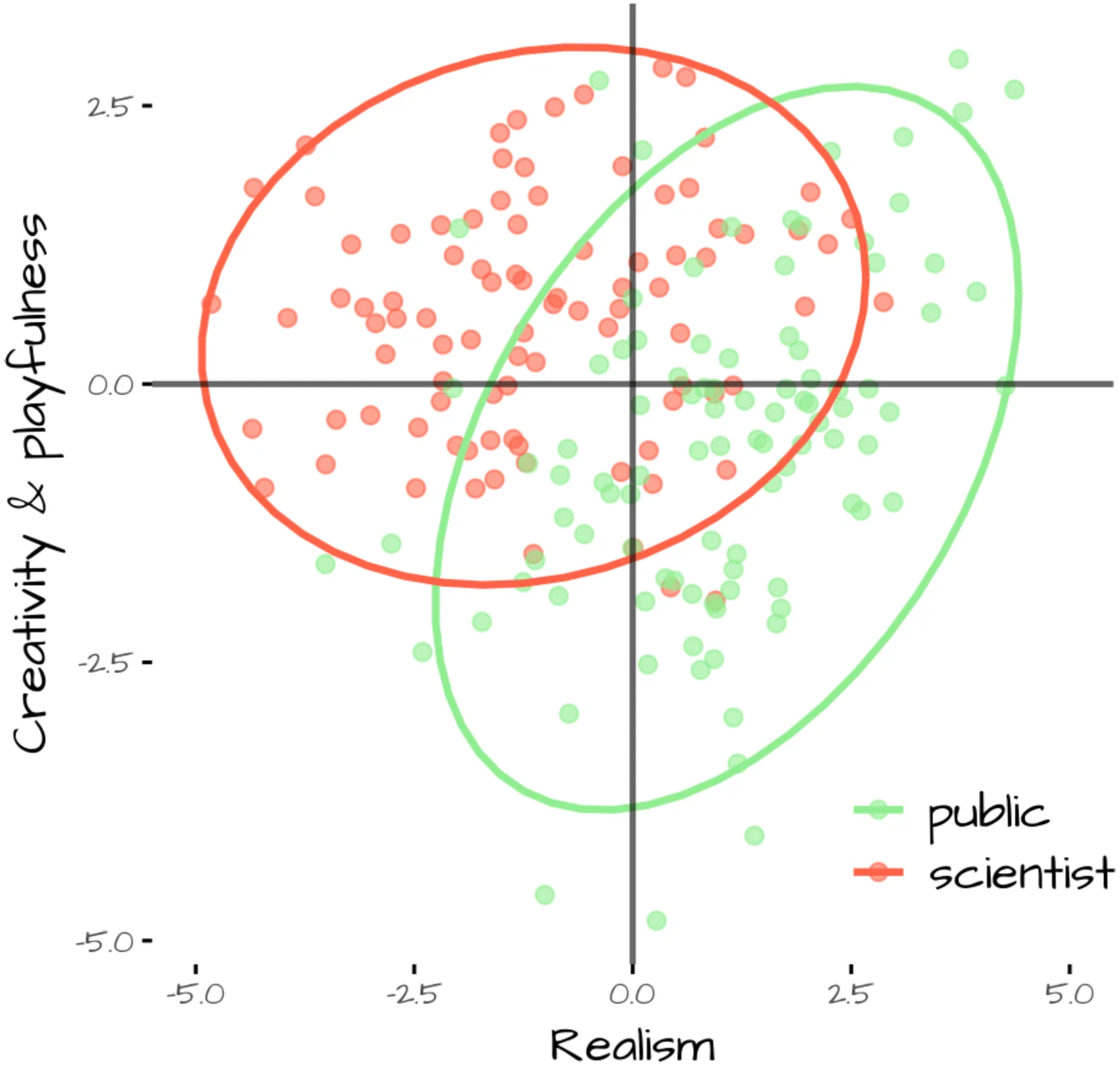
Participants from two groups (scientists versus public) along the two dominant dimensions identified by PCA.

## 4. Who agrees with the public?

When examining alignment between scientists’ response profiles and the average response profile of the public, we observed systematic variation across fields (table 3). Scientists in the biological sciences and physics showed the highest alignment with the public, whereas scientists in mathematics, social sciences and psychological sciences exhibited substantially lower alignment. Although these field-level comparisons are exploratory and based on modest sample sizes for most fields, the results suggest that scientific fields diverge from public conceptions of science to varying degrees. These differences may reflect variation in scientists’ epistemic commitments or differences in the public visibility of different sciences (e.g. high-school science teaching often relies on canonical examples from physics and biology). One relevant epistemic commitment is scientific realism. Natural scientists have previously been found to hold stronger realist views than social scientists [29], which could help explain the higher alignment of biologists and physicists with the public in our sample.

## 5. Why do scientists disagree?

We next examined whether demographic variables, such as age, gender and scientific field, systematically structure responses to different conceptions of science. Linear model analysis revealed small but consistent associations: across items, these variables explained a modest amount of variation in responses (median importance ranging from 0.01 to 0.30). Among them, scientific field accounted for the largest share of the explained variation (often greater than 50%), followed by age and then gender.

However, these effects did not translate into predictive power. When evaluated using cross-validation, out-of-sample $R^{2}$ values were consistently below zero (median $R^{2}=-0.22$, ranging from −0.47 to −0.06). In other words, knowing a scientist’s age, gender or field did not help predict how they would respond to these questions any better than simply guessing the average response. We observed the same pattern using a random forest model, which can capture more complex relationships than linear regression. Although this model also detected small associations between demographics and responses, it similarly failed to improve predictive accuracy (supplementary material, tables S3 and S4).

These results suggest that factors like age, gender and scientific field may only play a limited role in shaping how scientists think about science. In our dataset, these factors introduce weak detectable structure, which is not strong enough to support prediction on new data. Disagreement among scientists appears to arise largely within, rather than between, demographic groups.

Below, we discuss specific conceptualizations and reasons why scientists and the public agreed or disagreed with them. This qualitative analysis was exploratory and is intended to support and illustrate the quantitative results. Specifically, the authors read all open-ended explanations, discussed the themes that recurred in them, organized the results around these themes and selected illustrative quotes. The explanations were not formally coded.

## 6. Is science discovering reality or constructing it?

The key dimension of disagreement between scientists and the public identified by the PCA analysis concerns realism: whether science is understood as uncovering objective truth [88], or instead as actively constructing reality [6]. Notably, many contemporary philosophical positions occupy a middle ground between these views (e.g. [49,51]).

Both scientists and the public generally endorsed the idea that science is a ***search for truth***, although scientists were more reserved in their responses (96% of the public agreed, compared to 83% of scientists, p<0.001). Most members of the public made it clear that the search for truth is their main criterion of scientific activity: *‘[…] the point of science is to search for the truth’*, said the 35-year-old woman from Las Vegas, NV. By contrast, scientists often emphasized that the ‘truth’ science arrives at is limited and context-dependent: *‘It also depends on how we define truth, and whether this truth is absolute or contextual; unique or multiple; complete or partial. I would favor the latter options’*, explained an associate professor of complex systems.

In contrast to the broadly shared endorsement of science as truth-seeking, the idea that science ***constructs reality rather than discovers it*** received the weakest support among the public, but not among scientists (public: 19% agreed, 66% disagreed; scientists: 42% agreed, 38% disagreed, p<0.001). Public respondents frequently rejected this framing by appealing to the objectivity of scientific facts: *‘Science is based in fact and something that is indisputable so I don’t think it’s constructing a reality, but more proving it’*, explained the 30-year-old woman from Albany, NY. Scientists, by contrast, were more willing to entertain the idea that science constructs aspects of reality, though they interpreted this construction as operating within empirical constraints: *‘With constraints. Science does not aim to put forward random theories that don’t describe and/or predict reality. It is not the case that “anything goes.” But scientific theories do create narratives/worldviews, and we have and will continue to shift between, sometimes very different, schemes’*, clarified a PhD student in cognitive and information sciences.

Overall, when reflecting on the possibility of learning truth about the world, scientists were more sceptical than the public. Many scientists emphasized the limitations, assumptions and contingency of scientific results, whereas public respondents often articulated an idealized view of science as a direct route to objective truth.

## 7. Active versus passive

Many of the conceptualizations we probed differ in whether they grant scientists an active role in generating knowledge or instead portray them as passive readers of information that already exists in the world (i.e. representing versus intervening; [84]). Based on agreement distributions and participants’ justifications, we found that scientists tended to prefer more active conceptualizations, whereas members of the public also endorsed passive ones that view science as a unidirectional transfer of information from the world to scientists.

Both scientists and members of the public were generally supportive of conceptualizations emphasizing an active role of scientists, although levels of agreement depended on how strong scientists’ portrayed influence on knowledge was. Most respondents resonated strongly with viewing science as ***curiosity-driven exploration*** (99% of the public and 96% of scientists agreed) and as ***detective work*** (99% of the public and 92% of scientists agreed), conceptualizations that emphasize scientists’ active role in exploring and assembling information to find a solution.

Not all active conceptualizations elicited equally strong endorsement. For example, viewing science as an ***iterative dialogue with nature*** was more appealing to scientists than to the public (64% of the public and 84% of scientists agreed, p<0.05). As an associate professor of biology and medicine explained, *‘Nature is a mysterious conversation partner; it does not understand why we ask questions nor what we truly know; part of the art of science is learning which questions to ask.’* A 60-year-old man from Denver, CO disagreed, *‘A charming idea, but Nature is the one who does all the talking.’*

Another conceptualization emphasizing an active role for scientists is the idea that scientists may shape the very phenomena they study, captured by the metaphor of science as a ***tactile interaction with the world*** (figure 2; [48–50,74]). This view, interestingly, received lower agreement from scientists than from the public (73% of the public and 58% of scientists agreed), although the difference was not significant. Many members of the public emphasized the importance of measurement for science and often invoked observer effects in their justifications: *‘Touching reality is very apt because by measuring, humans experience natural phenomena and life itself firsthand, directly. Also because at the atomic level, the behavior of light varies depending on whether there is a conscious observer.’*, explained a 63-year-old woman from Pittsburgh, CA. Many scientists, on the other hand, were uncomfortable with the idea that their discipline exerts such an active influence on its subject matter.

In contrast to conceptualizations emphasizing the active role of scientists, those portraying science as a more passive endeavour received the lowest rates of agreement among scientists, but not among members of the public. One such example is the view of science as ***reading the book of nature*** (60% of the public and 30% of scientists agreed, p<0.01), one of the oldest Western conceptualizations of science, dating back to well before the enlightenment (e.g. Augustine of Hippo, fourth century AD). Historically, this conceptualization served as a bridge between religion (the book of God) and the emerging practice of systematically collecting knowledge about the world [57,63,64]. Despite its long history, this was the least endorsed conceptualization among scientists. Scientists particularly objected to the idea that knowledge about the world already exists, fully inscribed in nature, merely awaiting our reading. As one postdoc in philosophy of physics described, *‘I am unsure whether nature has a native language,’* and a PhD student in aerospace engineering suggested that *‘[science] is more like writing the book.’*

Another passive conceptualization that received relatively low agreement from scientists, but not from the public, was the idea of science as a ***mirror of nature*** (72% of the public and 42% of scientists agreed, p<0.001). This notion also has deep historical roots, with early Western encyclopedias even bearing the title *Mirror of Nature* (*Speculum Naturale*; *Speculum Maius* [67]). Scientists emphasized that science is not a passive reflection of the world, noting that an exact replica of reality would be of little use. Illustrating this view, a postdoctoral researcher in computer science explained, *‘The goal of science is to understand, i.e., identify structure in the components of reality, rather than merely to reflect them.’*

Consistent with this stance, the conceptualization of science as actively ***creating simplified representations of the world (e.g. a map of complex reality)***, rather than passively mirroring it, received substantially higher endorsement from scientists, but not from the public (67% of the public and 87% of scientists agreed, p<0.05). As a full professor of environmental social sciences noted, *‘Ideally, we would like to actually explain the world, but pragmatically, the best we can hope for is a simplified representation of […] it.’* By contrast, public respondents often rejected this framing, emphasizing the complexity of scientific knowledge: *‘Science is not a simplified representation of the world, but a rigorous, empirical, and complex representation of the world.’*, said a 34-year-old man from Cheney, KS, and *‘Science is often complicated and nuanced. It aims to understand, but that doesn’t make it simple.’*, explained a 45-year-old man from Lancaster, NY.

## 8. Incremental and planned versus disruptive and improvisational

Some of the probed conceptualizations portrayed science as an incremental activity, progressing through step-by-step improvements. Other conceptualizations instead presented science as a more disruptive and improvisational activity, with discovery being difficult to anticipate or plan in advance.

Thinking of science as ***construction work (building knowledge brick by brick)*** [58] or as ***problem-solving within paradigms*** [3,73] is very common. Consider moments when you have tried to identify and fill a ‘gap’ in the literature [89], or worried whether your work is sufficiently ‘foundational’ or adequately ‘builds on’ prior research. Surprisingly, both the construction work and problem-solving conceptualizations received more support from the public than from scientists (construction work: 87% public and 64% scientists agreed, p<0.001; problem-solving: 89% public and 64% scientists agreed, p<0.001).

While many scientists acknowledged that much of their work is incremental (e.g. adding new ‘bricks’ to a shared structure or solving problems within an existing paradigm), they also emphasized that science is not as cumulative or orderly as these conceptualizations suggest. Scientists frequently noted that science is not only about filling gaps, but also about actively creating new ones and, at times, demolishing the foundations of existing knowledge. Similarly, the problem-solving metaphor fails to capture how scientists often generate entirely new problems, overturn paradigms and redefine what counts as a useful or relevant question [3]. This tension was succinctly summarized by a professor of environmental social sciences, *‘This may be what the day-to-day work of most scientists is. I don’t think it’s necessarily the ideal, nor is it the complete story. We all want to be revolutionaries.’*

Reflecting this less directed side of science, some scientists endorsed the idea of science as ***creative improvisation*** (59% of scientists agreed) [72]. Those who agreed often still noted that this improvisational aspect of science coexists with more incremental, cumulative processes in which knowledge is indeed built ‘brick by brick’. By contrast, the emphasis on creativity was less appealing to members of the public (43% agreed to the improv conceptualization, p<0.05), and reactions were often emotionally charged. As a 47-year-old man from Somerville, MA put it, *‘There is very little room for creativity in science because you seek the truth about the universe, not make up something to explain it.’* Many others echoed this sentiment: *‘Science should be about facts only - no creative improv which can vary by scientist.’* (62-year-old man, Richmond, VA), and *‘You don’t make this up like in improv. It is based on rules and math.’* (41-year-old man, Pine City, NY).

To scientists, science is both cumulative and improvisational [90,91], but members of the public often interpret improvisation and creativity as signs of pseudoscience.

## 9. If science is a game, what game is it?

While respondents often converged on the main strengths and weaknesses of certain conceptualizations, others elicited a wide diversity of reasons for agreement or disagreement. One such case was the idea that science is like ***a game***, which received more support from scientists than from the public (43% of the public and 63% of scientists agreed, p<0.01), but was interpreted in substantially different ways.

Some scientists agreed with the game metaphor on the basis of shared emotional experience (*‘it is fun!’*, said a PhD student in psychology). Others described science as a form of play with, or even against, nature. For example, a research scientist in AI explained, *‘Guessing game as to what nature is like and like many games you can make the right move but still lose (chance is involved).’* Others emphasized that the social aspects of science are often gamified. A PhD student in economics offered the following perspective, *‘Science as such is not really like a game. The practice of science, however, which requires […] networking, strategizing about the topics and target journals […] very much is!’*

Scientists who disagreed with the game conceptualization emphasized that, unlike most games, science does not have a clearly defined objective or endpoint. Several also argued that science is more serious than a game. *‘More than a game, it has consequences.’*, said a professor of history. A professor of environmental social sciences added that, *‘thinking of science like a game trivializes the endeavor’*. These views echoed sentiments expressed by many members of the public, as summarized by a 39-year-old woman from Layton, Utah, *‘Science is not a game. It’s serious business.’*

## 10. Competition and power in science

Several of the conceptualizations framed science in explicitly social terms, emphasizing competition, conflict and power in scientific practice. Two such ideas—of science as a ***free marketplace of competing ideas*** and as a ***battle against ignorance***—elicited particularly polarized responses with bimodal agreement distributions by both groups.

Economics has contributed an influential conceptualization of science as a ***free marketplace of competing ideas*** [81,82]. This view was highly contested, with both the public and scientists showing bimodal agreement distributions (public: 58% agreed, 28% disagreed; scientists: 47% disagreed, 40% agreed; p<0.01). Some respondents endorsed this idealized picture, arguing that *‘there is no room for politics in science’* (professor of management), and that *‘In some sense, ideas are competing for resources and people are willing to pay more for ideas that are more important…, interesting or able to explain laws of nature.’* (PhD student in engineering). Many public respondents agreed: *‘Science in my mind does works like a marketplace of ideas, where theories compete, evidence sets the price and only the good ones survive’*, explained a 36-year-old man from Houston, TX.

Others emphasized how science often falls short of this ideal. *‘There are a lot of oligarchs and cartels.’*, said a professor of music. Some pointed to systematic biases: *‘Surely it’s irrational to expect the most influential ideas to somehow come from the countries with the most money…’*, noted a PhD student in psychological and behavioural sciences. Others highlighted unequal access to participation in science: *‘Unfortunately, there are structures in science that makes it that many people have long paths to be able to access the marketplace, while others were born just next to it…’*, explained a postdoc in biochemistry. Public respondents also emphasized financial conflicts of interest: *‘If only that were true! Recent scandals surrounding the irreproducibility of many experimental results, the involvement of government, and especially the involvement of private industries that have a financial interest in the conclusions drawn cast a long shadow over the idea of a “free” marketplace of ideas’*, emphasized 60-year-old man from Denver, CO.

The social dimensions of science and its relationship to the public came to the forefront when respondents reflected on whether science ***battles ignorance***, another conceptualization that proved controversial. Surprisingly, it was members of the public rather than scientists who resonated more strongly with this idea (public: 75% agreed, 18% disagreed; scientists: 47% agreed, 37% disagreed; p<0.001).

When explaining their agreement, members of the public frequently framed science as oriented towards truth. As a 47-year-old man from Somerville, MA put it, *‘When you are ignorant, you are not doing science because science is based to truth, not ignorance or lies.’* Others referred to contemporary political and societal contexts: *‘This is very true. There are some people that would rather be ignorant instead of reading scientific articles and discoveries to realize that they are wrong. For example, people that choose to remain ignorant about climate change and how it is caused by human activity.’* (25-year-old woman from Indianapolis, IN). Similarly, another respondent noted, *‘This seems more true than ever as we have people even within our own government who are very anti-science and want to let industry run roughshod over our environment.’* (51-year-old woman from Decatur, GA).

Despite these expectations that scientists should actively fight ignorance, several scientists pragmatically rejected this idea, suggesting that ‘battling ignorance’ is not part of their job. *‘People are free to be ignorant.’*, said a professor of history; *‘I’d prefer to ignore the ignorant, not fight them.’*, added a professor of management.

Other respondents, especially among scientists, challenged the idea that science provides a superior point of view. As a PhD student in aerospace engineering remarked, *‘science is a battle to make your particular form of ignorance respectable.’* Others emphasized the legitimacy of alternative ways of knowing. A research scientist in policy reflected, *‘I think that thinking that is what got us to where we are […] I do not like the words “battle” or “war” or “ignorance”. I know people who cannot read or write who are wiser than most people i know could be or ever will be.’* A 62-year-old man from Richmond, VA, echoed this concern, *‘I think scientists have gotten a bit arrogant and believe they are the only intelligent ones out there. They shouldn’t have a problem being questioned if they truly believe in science.’* Taken together, these responses rejected the aggressive framing and resisted placing science on a pedestal as the sole or superior way of understanding the world.

Finally, several scientists focused on the role ignorance plays within science, explaining that science progresses not by fighting ignorance but by embracing it [92]. As a professor of ecology and evolutionary biology explained, *‘Science embraces ignorance, done correctly. But it is willing to let go of bad ideas once they’ve been disproven or falsified.’* Similarly, a master’s student in physics noted that, *‘science is also about recognizing our ignorance and limits to our knowledge’*.

Taken together, these responses suggest that members of the public more often endorsed idealized framings of science as an unbiased and apolitical endeavour aimed at correcting everyone else’s ignorance. Scientists more frequently resisted these framings, emphasizing different sources of bias in science and the limits of scientific authority.

## 11. Discussion

Our survey presents a limited attempt to capture scientists’ and the public’s understandings of what science is. Even within our relatively limited samples, we were struck by the diversity of elicited perspectives. We observed substantial disagreement in how science was conceptualized, with scientists’ views appearing more heterogeneous and the public’s views more stereotyped. In general, the public tended to view science in an idealized way, almost as a machine for producing truths or a direct route to objective knowledge, with little room for politics and no place for creativity. Scientists, by contrast, were more reserved in their assessments. Almost no conceptualization received scientists’ full endorsement, and agreement was often accompanied by reservations and caveats. These caveats commonly concerned biases in the scientific process and the limitations of the scientific method.

There are multiple explanations for the disconnect between scientists’ and the public’s understandings of science. First, this result is consistent with prior work suggesting that lower levels of education are associated with lower epistemic vigilance or critical evaluation of knowledge claims [93–95], although this relationship is contested and was not supported by a recent meta-analysis of susceptibility to online misinformation [96]. Scientists’ higher levels of education (at least 70% of our scientist sample held a PhD) may foster a more critical stance towards all claims, including those about science itself. A second explanation concerns a mismatch between how science is taught and communicated to the public and the realities of scientific practice. This interpretation is partially supported by our finding that public conceptions of science were most strongly correlated with biologists’ and physicists’ views, representing fields that are most frequently portrayed as paradigmatic exemplars of scientific rigour.

Our findings resonate with a long tradition of work in science education on the ‘nature of science’, which has repeatedly documented that students and members of the public often hold idealized views of science, emphasizing objectivity and certainty [24–27]. However, this literature has typically approached such views compared to a limited set of philosophical reference points about what science ought to be. Therefore, disagreement among practising scientists has rarely been treated as a phenomenon of interest in its own right (except, e.g. [28,29]). By contrast, our results suggest that differences in how science is understood are not confined to science education and communication, but extend to scientists themselves, and that these differences may reflect distinct epistemic worldviews rather than simple misunderstandings of a single settled conception of science.

We speculate that the uncovered differences between public and scientists’ conceptualizations of science matter for science communication and education. The public’s idealized view of science [97] is consistent with research showing that most members of the public in many countries trust science and support its involvement in policymaking [98]. Our results, however, may be skewed by the left-leaning political ideology of Prolific participants, since left-leaning ideology is associated with higher trust in science in the USA [98]. One concern is that idealized views of science may set people up for disappointment when confronted with the realities of scientific practice, including uncertainty, disagreement and changes in conclusions as new evidence accumulates. If the public expects clear answers and definitive truths, then when scientists communicate disagreements among themselves or update their recommendations, this may be perceived as failure, potentially undermining trust [99].

The diversity within scientists’ understandings of science is itself worth closer examination. While some scientists described their work as creative and spontaneous, others emphasized routine and incremental aspects of their practice. Some expressed an idealistic view of science as a search for truth and a superior way of acquiring knowledge, whereas others stressed the limitations of science, highlighting how it often deviates from its ideals and proclaimed aspirations. It is possible that the abstract beliefs elicited in this study have little bearing on how scientists actually conduct their work. However, we speculate that these differing conceptualizations may shape scientists’ openness to different methodological reforms and science policy changes [47,100]. For instance, those who view science as a cumulative search for truth may be more inclined to support initiatives such as widespread replication, preregistration and stringent theory testing, seeing these as safeguards against introducing ‘wrong bricks’ into the scientific edifice. By contrast, scientists who see science as a creative enterprise, one among many ways of producing knowledge, may be more supportive of policies aimed at increasing the diversity of approaches, theories and people in science.

One open question is *why* scientists disagree so much about what science is, despite ostensibly doing the same job. One possibility is that scientists engage in fundamentally different kinds of research in different disciplines, which was only weakly supported by our results. Alternatively, scientists within disciplines can engage in different types of research, such as mathematical modelling or experimentation, with these roles shaping scientists’ conceptualization of science as a whole. Finally, it is likely that a shared understanding of science is simply assumed rather than explicitly taught or discussed (it is like ’the water we swim in’; [101]), leaving room for multiple interpretations of what the process is about. Indeed, the research on much simpler human concepts that are often explicitly taught, such as ‘penguin’ or ‘chicken’, demonstrates that even these enable a diversity of understandings, which is hypothesized to lead to noticeable societal effects [47].

The differences observed both between and within groups should be interpreted with caution, as respondents may understand the same statement in different ways. As illustrated in §9, there are many aspects of a game that might align or misalign with science. Moreover, people may interpret differently what they are being asked to judge, e.g. whether a statement concerns scientific data, theories, methods or activities. Members of the public may have interpreted many statements as being about scientific data or results, perhaps because that is how they most commonly encounter science, rather than through the scientific process itself. This could explain why public participants, but not scientists, tended to refer to ‘facts’. Another latent source of divergence is whether a statement is interpreted descriptively (how science is done) or normatively (how science should be done). We noticed this ambiguity in participants’ open-ended responses: some said, for example, that science should be like a curiosity-driven exploration yet in reality it often is not, or that science should be like a free marketplace but often isn’t. Such ambiguity could lead participants to give diverging responses even when they actually agree with each other. Future research could disentangle these sources of ambiguity, for instance by explicitly asking participants to evaluate statements descriptively or normatively, and about specific aspects of science such as data, theories or practices.

One possibility is that respondents make agreement judgments based on an overall sense of fit between a conceptualization and their view of science, and only subsequently articulate justifications for that judgment [102]. Importantly, even if agreement ratings themselves are somewhat ambiguous, the starkly opposing explanations elicited by some conceptualizations (e.g. claims that science leaves little room for creativity versus claims that creativity plays a central role) are less ambiguous and more clearly reflect genuine disagreement among respondents.

Our main analyses treat the public and scientists as two homogeneous groups. However, both our own results and broader research in the history, philosophy and sociology of science [16] suggest that this assumption is overly simplistic. Scientists differ substantially by discipline, role (e.g. experimentalist versus theorist), epistemic goals, educational tradition (e.g. Western versus non-Western training) and other dimensions [44]. Similarly, the public consists of people with very different attitudes towards science, cultural backgrounds, educational levels and political leanings, among other factors [103]. Collapsing this diversity into two contrasting groups of ‘scientists’ and ‘the public’ reflects common practice in experimental psychology, but it does not do justice to this rich composition, which may account for the within-group disagreement we observed, particularly among scientists. Notably, field, age and gender predicted individual scientists’ responses only weakly, suggesting that the most relevant sources of variation may lie elsewhere. Future research should aim to identify the structure that distinguishes different members of the public and different scientists, and to test whether this structure accounts for their understanding of science, or whether, conversely, their understanding of science shapes it (e.g. by influencing which educational and career paths people pursue).

Moreover, neither group was a representative sample of its respective population. The scientists were drawn mainly from visitors to the Santa Fe Institute, who are often invited because of their interdisciplinary and creative research. This may partly explain why the scientists in our sample emphasized the creative and subjective aspects of science. Similarly, the public sample was recruited through Prolific, whose US participant pool is not representative of the US population: Prolific participants tend to be younger, more highly educated and more politically liberal than the US population as a whole [104]. Because Prolific provides education and political affiliation only when they are used as screening criteria, we could not assess these characteristics directly.

Therefore, we see the current study as a seed for scaling up future research on how people conceptualize science. Expanding this line of work should involve engaging a larger and more diverse sample of scientists and the public, including scientists trained outside Western scientific traditions, and probing respondents with a broader range of different conceptualizations. The list of conceptualizations should be expanded on the basis of a more comprehensive review of the literature, and participants should be instructed more clearly whether to judge each conceptualization descriptively (what science is like) or normatively (what science should be), and whether it concerns the scientific process, theories or data. Collecting more data would allow us to better capture the variety of reasons respondents hold or reject particular ideas. Ideally, future work could enable more high-powered analyses of how these disagreements are related to scientists’ disciplinary backgrounds, types of research, career stages and other factors, as well as to the public’s cultural background, education and other differences.

The next time you disagree with a colleague or a member of the public about how science should be done, consider first asking: *what is science to you?*

## Supplementary Material



## Data Availability

The analysis code and anonymized data for the project are available at http://osf.io/ra4eg/overview?view_only=7e27bc4169254bb396b6c9559d35b086. Supplementary material is available online [105].

## Footnotes

^1^
Number of respondents after exclusions. We excluded 19 scientists who did not fully complete the survey and one Prolific participant who produced AI-generated responses.

^2^
The Likert scale was implemented as a drop-down menu with seven labelled options: ‘Strongly disagree’ (1), ‘Disagree’ (2), ‘Somewhat disagree’ (3), ‘Neither agree nor disagree’ (4), ‘Somewhat agree’ (5), ‘Agree’ (6) and ‘Strongly agree’ (7). In subsequent analyses, we classified responses of 5–7 as agreement with the statement and responses of 1–3 as disagreement; neutral responses (4) were counted in neither category.

^3^
The scientist sample was collected as part of a longer, multi-component study that prioritized efficient data collection and therefore did not require open-ended responses. After completing that study, we recognized the additional value of qualitative explanations and incorporated this component as mandatory in the public sample (which also served as an additional check against AI-generated responses).

^4^
However, since almost all items load in the same (positive) direction, this component also partly reflects participants’ overall tendency to agree with the conceptualizations.
