# Supplementary material for: Everyone knows what science is…until you ask
Source: R Soc Open Sci. 2026 Nov 11;13(11):260933. doi: 10.1098/rsos.260933 (PMC13750929; doi:10.1098/rsos.260933)
Supplement: Supplementary file 1 [file rsos.260933.zip › rsos.260933/rsos260933_si_001.pdf]

Supplementary Material for
"Everyone Knows What Science Is... Until You Ask"

Marina Dubova[1,*] and Qiawen Ella Liu[2]

[1]Santa Fe Institute, Santa Fe, NM 87501, USA
[2]Department of Computer Science, Princeton University, Princeton, NJ 08540, USA
[*]Corresponding author: mdubova@santafe.edu

October 3, 2026

Group differences in agreement with conceptualizations of science

Table S1 reports chi-square tests of independence comparing the agreement distributions of scientists and members of the public for each of the 18 conceptualizations of science. Chi-square tests are based on the full 2×7 contingency tables of agreement ($1 =$ strongly disagree to $7 =$ strongly agree) by group, with effect sizes reported using Cramér's V. Where expected cell counts were below 5, p-values were obtained via Monte Carlo simulation ($B = 20{,}000$ replicates). p-values are corrected for multiple comparisons using the Benjamini–Hochberg procedure. Three conceptualizations (Curiosity-driven Exploration, Flashlight, and Detective) elicited near-unanimous agreement in both groups, leaving no meaningful distributional variation to test; these are indicated by dashes.

Principal component loadings

To identify the dominant axes along which agreement profiles vary, we combined scientists and members of the public into a single dataset (retaining respondents missing $\leq 20\%$ of items, with the remaining missing values imputed by item-wise medians) and performed a principal component analysis on the standardized item-response matrix. The first principal component explained 20.8% of the variance, and the second explained 11.8%, for a cumulative 32.6%. Table S2 reports the loadings of each conceptualization on these two components. PC1 contrasts conceptions emphasizing the discovery of an external reality (positive loadings on Flashlight, Mirror, Detective, Book of Nature, Battle Against Ignorance, Search for Truth) with conceptions emphasizing the constructive aspects of science (negative loading on Constructing Reality). PC2 separates framings emphasizing creative and playful inquiry (Improv, Game, Beauty and Elegance, Constructing Reality, Simplified Representations) from framings emphasizing accurate mirroring and truth-seeking (negative loadings on Search for Truth, Flashlight, Mirror). A logistic regression classifier trained on participants' PC1 and PC2 scores predicted group membership (scientist vs. public) with 82.1% leave-one-out cross-validated accuracy.

Demographic structure of agreement among scientists

To assess whether scientists' demographic characteristics (age, gender, broad field) systematically structure their agreement with each conceptualization of science, we fit a separate random

Table S1: Chi-square tests comparing scientists and members of the public on agreement distributions for different conceptualizations of science. Effect sizes are reported using Cramér's V. p_{FDR} values are corrected for multiple comparisons using the Benjamini–Hochberg procedure. Conceptualizations with response categories that were empty in both groups, for which chi-square tests are undefined, are indicated by dashes. Where expected cell counts were low, p-values were obtained using Monte Carlo–simulated chi-square tests.

Conceptualization	n_{Sci}	n_{Pub}	χ^2	V	p	p_{FDR}
SEARCH FOR TRUTH	92	99	35.28	0.43	5.0×10^{-5}	1.5×10^{-4}
PROBLEM SOLVING	92	98	32.64	0.41	5.0×10^{-5}	1.5×10^{-4}
CONSTRUCTION WORK	92	99	31.98	0.41	5.0×10^{-5}	1.5×10^{-4}
MIRROR	92	95	31.11	0.41	5.0×10^{-5}	1.5×10^{-4}
CARVING NATURE	92	95	27.21	0.38	5.0×10^{-5}	1.5×10^{-4}
CONSTRUCTING REALITY	92	95	24.91	0.37	1.0×10^{-4}	2.5×10^{-4}
BATTLE AGAINST IGNORANCE	92	97	24.77	0.36	3.8×10^{-4}	8.1×10^{-4}
BOOK OF NATURE	92	96	21.70	0.34	1.4×10^{-3}	2.6×10^{-3}
BEAUTY AND ELEGANCE	92	97	20.99	0.33	1.8×10^{-3}	3.1×10^{-3}
GAME	92	98	18.88	0.32	4.4×10^{-3}	6.0×10^{-3}
FREE MARKETPLACE	92	98	18.85	0.31	4.4×10^{-3}	6.0×10^{-3}
IMPROV	92	95	16.01	0.29	1.4×10^{-2}	1.7×10^{-2}
SIMPLIFIED REPRESENTATIONS	92	96	13.00	0.26	3.8×10^{-2}	4.0×10^{-2}
DIALOGUE WITH NATURE	92	95	12.86	0.26	3.4×10^{-2}	3.9×10^{-2}
TOUCHING REALITY	92	97	6.81	0.19	0.35	0.35
CURIOSITY-DRIVEN EXPLORATION	92	99	–	–	–	–
FLASHLIGHT	92	99	–	–	–	–
DETECTIVE	92	99	–	–	–	–

Table S2: Principal component loadings for the first two components. Positive and negative loadings indicate the direction and strength with which each conceptualization contributes to the component.

Conceptualization	PC1	PC2
FLASHLIGHT	0.356	-0.081
MIRROR	0.332	-0.077
DETECTIVE	0.311	-0.061
BOOK OF NATURE	0.304	0.153
BATTLE AGAINST IGNORANCE	0.303	-0.043
SEARCH FOR TRUTH	0.296	-0.200
CURIOSITY-DRIVEN EXPLORATION	0.277	0.043
CONSTRUCTION WORK	0.265	0.069
FREE MARKETPLACE	0.264	0.138
PROBLEM SOLVING	0.237	-0.048
TOUCHING REALITY	0.212	0.045
CARVING NATURE	0.208	0.069
DIALOGUE WITH NATURE	0.081	0.285
BEAUTY AND ELEGANCE	0.068	0.381
SIMPLIFIED REPRESENTATIONS (MAPS)	0.059	0.343
IMPROV	0.042	0.447
GAME	-0.037	0.398
CONSTRUCTING REALITY	-0.125	0.426

forest regression model for each conceptualization, with the rating as the outcome and age, gen-

der, and field as predictors. Permutation importance for each predictor was estimated, and 95% confidence intervals and the proportion of bootstrap samples in which importance was strictly positive were obtained from 500 bootstrap resamples of the participants. The single respondent identifying as "Complex Systems" was excluded from the field predictor because of unique-category sparsity. Table S3 reports per-conceptualization bootstrap permutation importance. Across conceptualizations, scientific field carried the largest median importance, followed by age and then gender; for the great majority of conceptualizations, the proportion of bootstrap samples with positive importance exceeded 0.95 for all three predictors, indicating reliably non-zero (if modest) demographic structure.

To make these importances comparable across conceptualizations, Table S4 reports the same bootstrap distributions normalized by the total positive importance within each sample (so that age, field, and gender shares sum to 1 within a sample). Across all 18 conceptualizations, scientific field accounted for a median of 45.3% of the explained importance (range across conceptualizations: 37.1–52.8%), age for 31.1% (23.1–41.7%), and gender for 21.5% (16.5–31.2%).

We also fit linear regressions predicting each rating from age, gender, and field, and computed predictor partial R^2. The total explained variance per conceptualization (sum of partial R^2 across predictors) was modest, ranging from 0.11 to 0.39 (median 0.20). Critically, when out-of-sample predictive accuracy was assessed via 10-fold cross-validation (5 repeats), cross-validated R^2 values were uniformly negative (median $R^2 = -0.22$, range -0.47 to -0.06), indicating that knowing a scientist's demographic profile does not improve prediction of their agreement above the marginal mean. The same pattern held for the random forest model. Together, these analyses indicate that age, gender, and field introduce small, statistically detectable but practically weak structure in scientists' responses, and that disagreements among scientists about how to conceptualize science arise largely within, rather than between, demographic groups.

Table S3: Random Forest analysis. Bootstrap permutation importance of demographic variables by conceptualization. Values show the median permutation importance across bootstrap samples, with 95% bootstrap confidence intervals and the proportion of bootstrap samples in which importance was positive.

Conceptualization	Predictor	Median	2.5%	97.5%	$P(\text{importance} > 0)$
SIMPLIFIED REPRESENTATIONS (MAPS)	Age	0.155	0.042	0.395	1.000
	Field	0.205	0.022	0.618	0.986
	Gender	0.135	−0.001	0.399	0.970
BOOK OF NATURE	Age	0.465	0.169	0.829	1.000
	Field	0.549	0.135	1.090	1.000
	Gender	0.252	0.044	0.602	0.994
TOUCHING REALITY	Age	0.505	0.251	0.840	1.000
	Field	0.505	0.185	0.951	1.000
	Gender	0.322	0.056	0.679	0.998
PROBLEM SOLVING	Age	0.362	0.135	0.716	1.000
	Field	0.467	0.152	0.900	1.000
	Gender	0.209	0.009	0.542	0.984
BATTLE AGAINST IGNORANCE	Age	0.521	0.180	1.110	1.000
	Field	0.906	0.399	1.690	1.000
	Gender	0.415	0.133	0.934	1.000

Conceptualization	Predictor	Median	2.5%	97.5%	$P(\text{importance} > 0)$
CARVING NATURE	Age	0.323	0.132	0.609	1.000
	Field	0.558	0.173	1.020	1.000
	Gender	0.250	0.017	0.640	0.982
CURIOSITY-DRIVEN EXPLORATION	Age	0.080	0.014	0.201	0.994
	Field	0.185	0.047	0.366	0.998
	Gender	0.066	−0.029	0.439	0.906
FREE MARKETPLACE	Age	0.560	0.217	1.120	1.000
	Field	0.662	0.256	1.250	1.000
	Gender	0.511	0.183	1.080	1.000
BEAUTY AND ELEGANCE	Age	0.391	0.126	0.789	1.000
	Field	0.579	0.183	1.120	1.000
	Gender	0.288	0.013	0.758	0.984
SEARCH FOR TRUTH	Age	0.247	0.086	0.555	1.000
	Field	0.479	0.176	1.100	1.000
	Gender	0.173	0.023	0.530	0.988
DIALOGUE WITH NATURE	Age	0.266	0.100	0.566	1.000
	Field	0.355	0.102	0.813	1.000
	Gender	0.288	0.079	0.750	0.996
FLASHLIGHT	Age	0.312	0.115	0.639	1.000
	Field	0.614	0.226	1.190	1.000
	Gender	0.347	0.099	0.716	0.998
GAME	Age	0.506	0.175	1.110	1.000
	Field	0.644	0.208	1.290	1.000
	Gender	0.241	−0.067	0.769	0.930
MIRROR	Age	0.694	0.404	1.100	1.000
	Field	1.020	0.484	1.770	1.000
	Gender	0.449	0.159	0.965	1.000
CONSTRUCTION WORK	Age	0.241	0.032	0.594	0.992
	Field	0.406	0.108	0.918	0.996
	Gender	0.150	−0.068	0.447	0.882
IMPROV	Age	0.299	0.059	0.689	0.992
	Field	0.441	0.115	0.930	0.996
	Gender	0.148	−0.120	0.623	0.822
DETECTIVE	Age	0.141	0.027	0.428	1.000
	Field	0.264	0.041	0.776	0.998
	Gender	0.163	0.004	0.460	0.986
CONSTRUCTING REALITY	Age	0.575	0.253	1.080	1.000
	Field	0.577	0.145	1.100	0.998
	Gender	0.222	0.021	0.544	0.988

Table S4: Random Forest analysis. Relative importance of demographic variables by metaphor. Values show median relative importance (share of total positive demographic importance) with 95% bootstrap confidence intervals.

Metaphor	Predictor	Median	2.5%	97.5%
SIMPLIFIED REPRESENTATIONS (MAPS)	Age	0.321	0.142	0.612
	Field	0.411	0.103	0.685
	Gender	0.256	0.000	0.506
BOOK OF NATURE	Age	0.369	0.202	0.575
	Field	0.423	0.187	0.619
	Gender	0.199	0.048	0.382
TOUCHING REALITY	Age	0.378	0.241	0.578
	Field	0.371	0.233	0.536
	Gender	0.246	0.077	0.360
PROBLEM SOLVING	Age	0.351	0.217	0.542
	Field	0.441	0.245	0.641
	Gender	0.197	0.019	0.379
BATTLE AGAINST IGNORANCE	Age	0.275	0.129	0.442
	Field	0.488	0.310	0.676
	Gender	0.225	0.091	0.418
CARVING NATURE	Age	0.283	0.160	0.509
	Field	0.477	0.270	0.709
	Gender	0.223	0.033	0.393
CURIOSITY-DRIVEN EXPLORATION	Age	0.231	0.061	0.520
	Field	0.525	0.221	0.851
	Gender	0.207	0.000	0.570
FREE MARKETPLACE	Age	0.318	0.181	0.494
	Field	0.378	0.214	0.584
	Gender	0.289	0.148	0.486
BEAUTY AND ELEGANCE	Age	0.304	0.160	0.494
	Field	0.453	0.257	0.694
	Gender	0.232	0.031	0.401
SEARCH FOR TRUTH	Age	0.267	0.138	0.464
	Field	0.528	0.345	0.766
	Gender	0.193	0.035	0.342
DIALOGUE WITH NATURE	Age	0.292	0.163	0.515
	Field	0.390	0.204	0.563
	Gender	0.312	0.152	0.463
FLASHLIGHT	Age	0.247	0.133	0.387
	Field	0.477	0.281	0.689
	Gender	0.269	0.093	0.468
GAME	Age	0.367	0.172	0.621
	Field	0.453	0.226	0.752
	Gender	0.165	0.000	0.344
MIRROR	Age	0.320	0.215	0.512

Metaphor	Predictor	Median	2.5%	97.5%
	Field	0.465	0.298	0.628
	Gender	0.207	0.091	0.365
CONSTRUCTION WORK	Age	0.296	0.092	0.572
	Field	0.504	0.264	0.860
	Gender	0.174	0.000	0.415
IMPROV	Age	0.322	0.114	0.577
	Field	0.481	0.217	0.824
	Gender	0.165	0.000	0.410
DETECTIVE	Age	0.242	0.101	0.519
	Field	0.452	0.206	0.734
	Gender	0.279	0.029	0.494
CONSTRUCTING REALITY	Age	0.417	0.226	0.710
	Field	0.404	0.169	0.622
	Gender	0.166	0.026	0.307

Correlation structure of agreement within each group

To complement the network visualization in the main text, we computed the full pairwise Kendall correlation matrix between agreement responses to the 18 conceptualizations of science, separately for members of the public and for scientists. Ratings were valence-coded prior to computation (1–3 $\rightarrow\ -1$; 4 $\rightarrow\ 0$; 5–7 $\rightarrow\ +1$). Figure S1 shows the resulting heatmaps, with rows and columns ordered identically in both panels (by hierarchical clustering of the merged-group correlation matrix using $1-\tau$ as the distance metric and average linkage). Figure S2 shows the entrywise difference (scientists $-$ public).

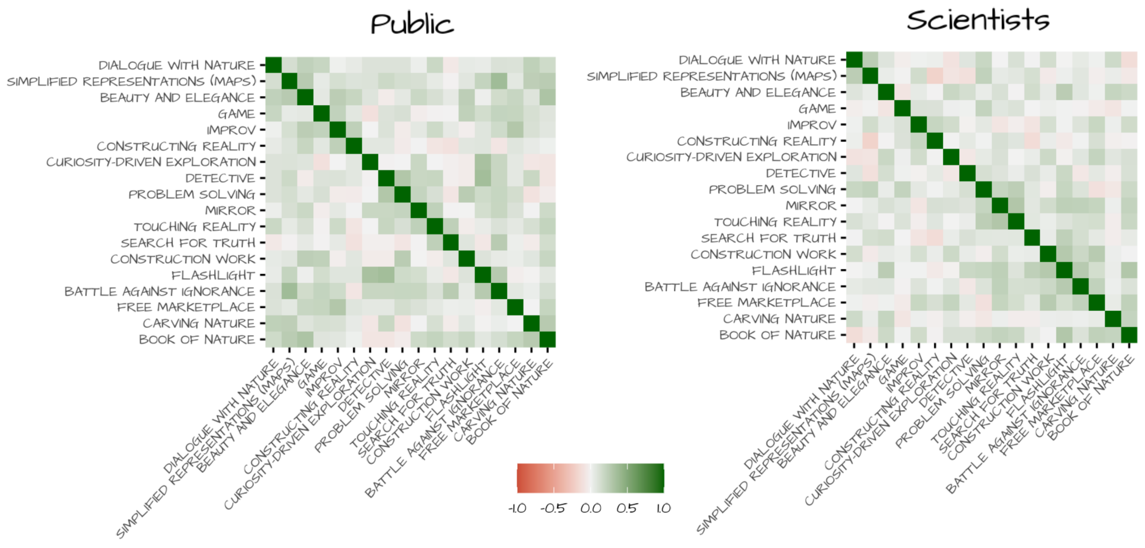

Figure S1: **Correlation structure of responses within each group.** Heatmaps show pairwise Kendall correlations between responses to the 18 conceptualizations of science for members of the public (left) and scientists (right). Color indicates correlation strength and sign (positive vs. negative). Questions are ordered identically in both panels to facilitate comparison.

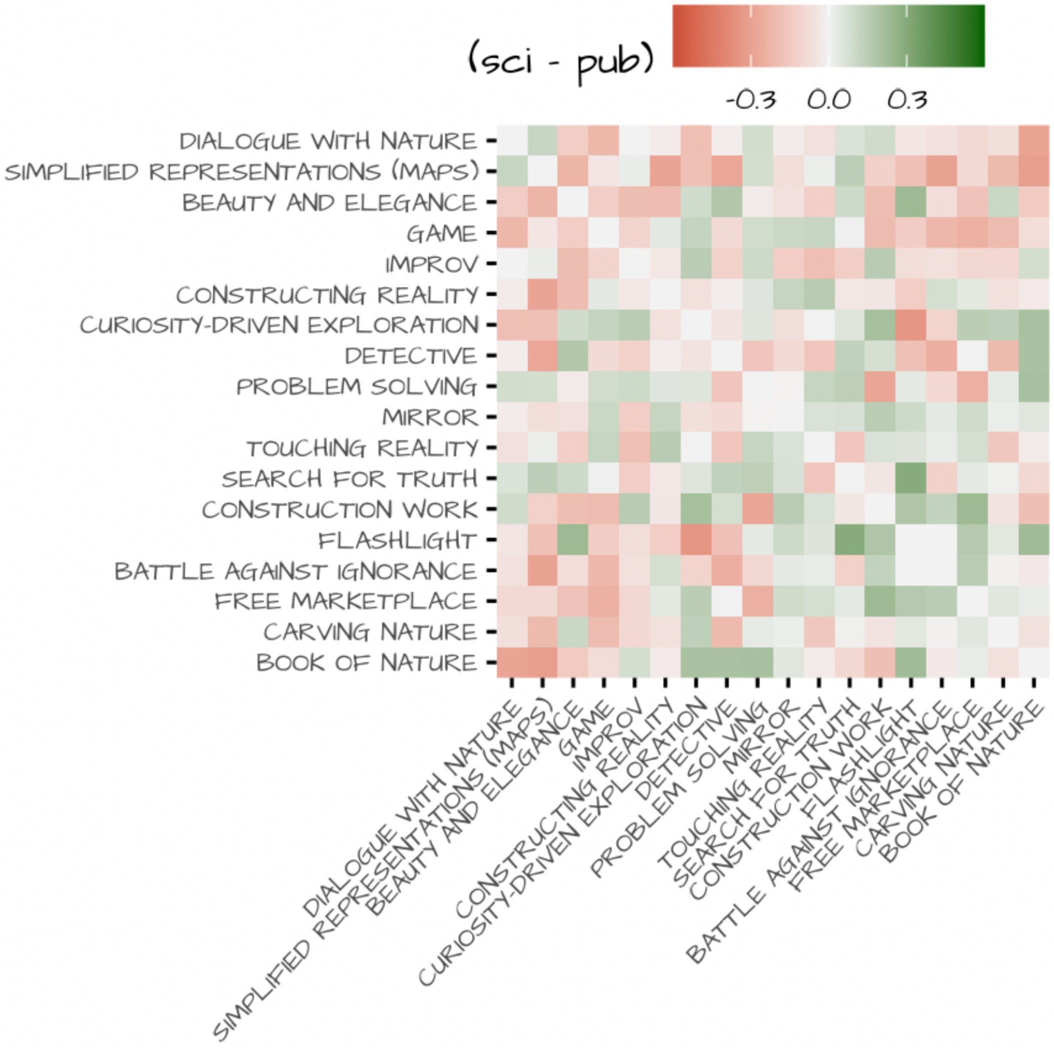

Figure S2: **Between-group differences in correlation structure (Public−Scientists).** Heatmap shows the difference in pairwise Kendall correlations between members of the public and scientists for each pair of conceptualizations. Positive values (green) indicate stronger positive association among public respondents than among scientists; negative values (red) indicate stronger association among scientists than among public respondents. Question ordering matches Figure S1.
